# Implementation of leakage elimination operators and subspace protection

**DOI:** 10.1038/s41598-020-75730-1

**Published:** 2020-11-02

**Authors:** B. G. Markaida, L.-A. Wu

**Affiliations:** 1 Department of Theoretical Physics and History of Science, The Basque Country University (UPV/EHU), P.O. Box 644, 48080 Bilbao, Spain; 2 Ikerbasque, Basque Foundation for Science, 48011 Bilbao, Spain

**Keywords:** Physics, Quantum physics

## Abstract

Decoherence-induced leakage errors can potentially damage physical or logical qubits embedded in a *subspace* of the entire Hilbert space by coupling them to other system levels. Here we report the first experimental implementation of Leakage Elimination Operators (LEOs) that aims to reduce this undermining. LEOs are a type of dynamical decoupling control that have been previously introduced to counteract leakage from a *chosen* subspace into the rest of a Hilbert space, and have been widely explored theoretically. Different from other error correction strategies, LEOs are compatible with any gate sequence in a code space, and thus, compatible with universal quantum computation. Using IBM’s cloud quantum computer (QC), we design three potentially applicable examples of *subspaces* in two- and three-qubit Hilbert spaces and derive the explicit forms of the corresponding LEOs for these subspaces. For the first time, we experimentally demonstrate that these LEOs significantly suppress leakage. The results also show that the LEO time-scale condition can be satisfied with noise in the IBM’s cloud QC and pave a way for quantum setups to get rid of leakage trouble.

## Introduction

Quantum computation relies on qubits, the fundamental quantum information units. For robustness and error correction purposes, it is beneficial to encode the available physical qubits into *logical qubits*, forming the logical qubit subspace. Ideally, no information would be lost from this subspace into the rest of the qubit system or the environment; however, whenever we deal with a real quantum system, we will encounter non-unitary system dynamical processes arising from system-environment coupling, referred to as *decoherence*. Such decoherence results in a loss of information from the system to the environment, as well as between subspaces of the system. This mixing between subspaces is called *leakage*, and it is a source of severe errors in quantum computing. Decoherence contributes to the decay of quantum coherence and entanglement, which are crucial in quantum computing and control^1^, and, particularly, leakage errors destroy the error protection benefit that we would expect from an encoding of the qubits^2,3^. It is, therefore, unsurprising that decoherence is regarded as the biggest challenge nowadays in quantum information, technologies and quantum computation in particular.

Numerous efforts have been made to overcome the hardships that decoherence produces, both through a quantum circuit model point of view and in the realm of equivalent adiabatic quantum computation^4–7^. These efforts include quantum error correction codes (QECCs)^8^, error avoiding and noiseless quantum codes or decoherence-free subspaces (DFS)^1,9–14^, dynamical decoupling^15,16^, getting rid of decoherence in solid-state quantum devices by encoding^17^. Among different types of decoherence, the particularly deleterious leakage errors in quantum information processing aroused our special attention^2^. Over the years, theories based on Leakage Elimination Operators have been perfectly developed and applied in various theoretical aspects of quantum information processing and quantum control dynamics^18^, for instance, strategies to improve quantum adiabatic processes^19,20^, control of decoherence through randomized white noise fields^21^. Therefore the next crucial step is to experimentally test the feasibility of these theoretical predictions and proposals.

In this paper, we consider three potentially applicable examples of *subspaces* in two- and three-qubit Hilbert spaces and study the feasibility of leakage suppression via *leakage elimination operators* (LEOs) on IBM’s cloud quantum computer. For the first time, we implement LEOs on the IBM’s QC^15^ and experimentally confirm that the LEOs can significantly suppress leakage, as predicted in the previous theory^2,3,22^. While other error-correction protocols such as QECCs are not necessarily compatible with all encodings, the LEO commutes with all operations on the code space and is compatible with universal quantum computation^2^, which allows their application alongside any algorithm running in a QC. On the other hand, a universal large-scale fault-tolerant quantum computer (QC) is still far from reach, due to complications in experimental implementations of the aforementioned logical qubit-encoding and stabilizers. Most research is thus focused on the application of quantum theory to quantum setups and quantum algorithms, which are of interest for building a future large-scale fault-tolerant QC. Therefore, this study also paves a way for generic use in these quantum setups to dispose of leakage troubles.

## Universal leakage elimination

Efforts to get rid of decoherence begin with the choice of the logical qubit subspace. If one could, somehow, find an available subspace that remains protected against decoherence, a *decoherence-free subspace*, that would constitute the most adequate choice for the logical qubit subspace. However, such a passive idealization is difficult to find, so typically, one looks for active ways to prevent decoherence. In this work, we aim to make the logical qubit subspace *leakage-free*, that is, we try to suppress leakage from the encoded subspace, or *codespace*, $\mathcal {C}$, to other states of the Hilbert space which reside in the orthogonal complement of the codespace, $\mathcal {C}^{\perp }$^3^, such as in continuous variable systems^23^. Elimination of the coupling between these subspaces rids the system from the most pressing decoherence-induced error. Further protection from decoherence, if necessary, could be achieved through, for instance, quantum error-correction codes (QECCs). To obtain these *leakage-free subspaces*, we will apply leakage-elimination operators, and we will do this through dynamical decoupling sequences.

Leakage elimination operators are based on dynamical decoupling controls. These are control pulses used to average away noise in quantum systems, and can be understood as a projection onto a subspace of the space of operators acting on the system Hilbert space $\mathcal {H}_{S}$^3^. More specifically, we will benefit from the application of fast, intense *bang-bang* (BB) pulses. The briefness and strength of these pulses effectively reduce the system-bath interaction Hamiltonian. Consider a general Hamiltonian,

1 $$\begin{aligned} H=H_{S}+H_{B}+H_{SB}, \end{aligned}$$

with $H_{S}$ ($H_{B}$) acting exclusively on the system (bath) and $H_{SB}$, the *system-bath interaction Hamiltonian* coupling the system to the bath. If we are to apply the dynamical decoupling method, that means we will be applying control pulses periodically to our system, leaving an interval $\Delta t$ of free evolution between them. Denoting these control pulses as $U_{i}$, and considering free evolution to be negligible while the control pulses are acting on the system, we obtain an expression for an effective unitary evolution for the combined system-bath after *N* control pulses^3^,

2 $$\begin{aligned} U_{\text {eff}}\approx \prod _{i=0}^{N-1}U_{i}\exp {[-iH\Delta t]}U_{i}^{\dagger }. \end{aligned}$$

The control sequences should be applied as fast as possible, and the propagator (2) will be exact when $N\rightarrow \infty$ and $\Delta t\rightarrow 0$. The first order $U_{\text {eff}}$ is described by an effective Hamiltonian,

3 $$\begin{aligned} H_{\text {eff}}\approx \frac{1}{N}\sum _{i=0}^{N-1}U_{i}HU_{i}^{\dagger }. \end{aligned}$$

Ideally, we would be able to completely eliminate the system-bath interaction for $N\rightarrow \infty$. The closer our experimental realization is to an ideal BB pulse scenario, the more efficient our LEO will be. This effectiveness depends mainly on three factors: pulse strength, pulse duration, and the time interval between pulses. The higher the pulse strength and the shorter the time scales, the closer the pulse will be to an ideal BB pulse. More precisely, the effectiveness of an LEO depends on the *integral* of these pulse sequences^24^, which can be of great interest when considering non-ideal LEOs. However, in this work, we will consider our BB sequences to be ideal.

To construct an LEO, we arrange the basis vectors of a *N*-level Hilbert space $\mathcal {H}_{N}$ and assume the first two levels, $|0\rangle$ and $|1\rangle$, correspond to our logical qubits. Then, as is stated in^2^, we can easily classify all system operators into (a) logical operators *E* acting on the qubit subspace $\mathcal {C}$, (b) operators acting on the orthogonal complement of this subspace, that is, acting on $\mathcal {C}^{\perp }$, that thus act entirely outside of the logical qubit subspace, and (c) leakage operators *L*, that connect these two orthogonal subspaces, and that take the following forms:

4 $$\begin{aligned} \begin{array}{ccc} E=\begin{pmatrix} B &{} 0 \\ 0 &{} 0 \end{pmatrix}, &{} E^{\perp }=\begin{pmatrix} 0 &{} 0 \\ 0 &{} C \end{pmatrix}, &{} L=\begin{pmatrix} 0 &{} D \\ F &{} 0 \end{pmatrix}. \end{array} \end{aligned}$$

Here *B*, *C*, *D* and *F* are blocks of dimensions $2\times 2$, $(N-2)\times (N-2)$, $2\times (N-2)$ and $(N-2)\times 2$, respectively. This decomposition is valid both for physical and logical qubits. The general form of an LEO is^2^,

5 $$\begin{aligned} R_{L}=e^{i\varphi }\begin{pmatrix} -I &{} 0 \\ 0 &{} I \end{pmatrix}, \end{aligned}$$

where the identity blocks *I* have the dimension of the subspace of the encoded qubits and its orthogonal subspace. That is, the dimension of these blocks matches the ones of the blocks in (4). $\varphi$ is a global phase. This operator satisfies the following commutation and anti-commutation relations:

6 $$\begin{aligned} \begin{array}{cc} [R_{L},E]=[R_{L},E^{\perp }]=0,&\{R_{L},L\}=0 \end{array} \end{aligned}$$

Since the LEO $R_{L}$ commutes with all operators that act on the encoded qubit subspace, we conclude that LEOs can be applied alongside any logical operation and are, thus, compatible with universal quantum computing. After applying a BB parity-kick sequence, we have

7 $$\begin{aligned} \lim _{m\rightarrow \infty }&\left( e^{-iH_{\text {SB}}t/m}R_{L}^{\dagger }e^{-iH_{\text {SB}}t/m}R_{L}\right) ^{m} \nonumber \\&=\lim _{m\rightarrow \infty }\left( e^{-iH_{\text {SB}}t/m}e^{-iR_{L}^{\dagger }H_{\text {SB}}R_{L}t/m}\right) ^{m} \nonumber \\&=e^{-iH_{E}t}e^{-iH_{E^{\perp }}t}, \end{aligned}$$

where $H_{\text {SB}}=H_{E}+H_{E^{\perp }}+H_{L}$ is the system-bath interaction. Here $H_{E}$ and $H_{E^{\perp }}$ correspond to the Hamiltonians acting on the qubit subspace and its orthogonal subspace, respectively, and $H_{L}$ is the Hamiltonian of leakage operators. Note that, taking into account the relations in (6), and knowing that each of the terms of the Hamiltonian will be composed of operators as the ones described in (4) the term $e^{-iR_{L}^{\dagger }H_{\text {SB}}R_{L}t/m}$ becomes $e^{-i(H_{E}+H_{E^{\perp }}-H_{L})t/m}$. The term $e^{-iH_{E^{\perp }}t}$ in (7) acts outside the logical qubit subspace and will thus have no effect on the code of interest. The term $e^{-iH_{E}t}$ does act on the logical qubit subspace and can therefore be the source of logical errors. If necessary, these errors may need further treatment, either by QECCs or additional BB pulses^3^. We see, however, that the leakage $H_{L}$ has been eliminated. For practical purposes, we need only consider $m=1$, and Eq. (7) will hold up to order $t^2$. The condition $t\ll 1/\omega _{c}$ must also be fulfilled, where $\omega _{c}$ is the bath high-frequency cutoff^2^.

A general choice for LEOs is

8 $$\begin{aligned} R_{L}=\exp {(\pm i\pi \hat{n}\cdot \vec {\sigma }P)}, \end{aligned}$$

where $\vec {\sigma }$ is a vector containing all three Pauli matrices (or *X*, *Y* and *Z* logical operations), $\hat{n}$ is a real unit vector, and *P* is a projector operator onto the qubit subspace. When a canonical logical operation is available, however, this projector becomes redundant and we may write an LEO as in^3^:

9 $$\begin{aligned} R_{L}=\exp (-i\pi \sigma _{L}), \end{aligned}$$

where $\sigma _{L}$ is any operation fulfilling $\sigma _{L}^{\dagger }=\sigma _{L}$, $\sigma _{L}^{2}=I$, and $\sigma _{L}|\psi \rangle =0$ for any $|\psi \rangle \in \mathcal {C}^{\perp }$.

## Methodology

We have experimentally tested three LEOs using IBM’s cloud quantum computer. The IBM Q project offers access to several quantum devices; in particular, we have worked with the five-qubit IBMX2 server in Yorktown (see^25^ for technical specifications)—as well as the QASM simulator in the same platform, for testing the code before sending it to the server. The native gates in IBMQ are single qubit rotation $R_{\alpha }(\phi )=\exp {(i\phi /2\sigma _{\alpha })}$, with $\alpha =\{x,y,z\}$ and $\sigma _{\alpha }$ being the Pauli matrices. In practice, we have used *X* ($\sigma _{x}$), *Z* ($\sigma _{z}$) and *H* (Hadamard gate) as single-qubit gates and the two-qubit CNOT (controlled-*X*) gate. For each program, the computer makes 1024 shots. The programs have been written in qiskit^26^, using the notebooks provided by IBM’s platform. See^27,28^ for a introductory tutorials on qiskit.

For testing the effectiveness of each LEO, we have proceeded as follows: We first initialize the system to be in a quantum state corresponding to the leakage-free subspace of interest. Then we study the effect of the LEO in the system. For that purpose, we apply the corresponding LEO *N* times, and measure. Similarly, to study the free evolution of the same state, we apply *N* identity gates to a system initialized in the same way. We repeat this process for $N\in [1,600]$. Each experiment was then repeated inserting an identity gate between LEO pulses, to study the effect of increased free-evolution intervals in the effectiveness of leakage elimination. In summary, we took the following steps:

1. Initialization of the system.
2. Evolution of the system for $N\in [1,600]$ pulses:
  - Under application of LEO
    i. With the smallest possible time interval between pulses.
    ii. With an extra identity gate between pulses.
  - Under free evolution
    i. With the smallest possible time interval between pulses.
    ii. With an extra identity gate between pulses.
3. Measurement.

We have tested whether the subspaces $\mathcal {S}_{1}=\Vert 01\rangle ,|10\rangle \}$, $\mathcal {S}_{2}=\{|{001}\rangle , |{010}\rangle , |{100}\rangle , |{111}\rangle \}$ and $\mathcal {S}_{3}=\{|{10}\rangle -|{11}\rangle \}$ are decoherence-free against collective dephasing, for three-site quantum state transfer^29^ and eigenvectors of the CNOT gate. For each of these subspaces we have used the appropiate LEO por protection from leakage.

**Figure 1:**
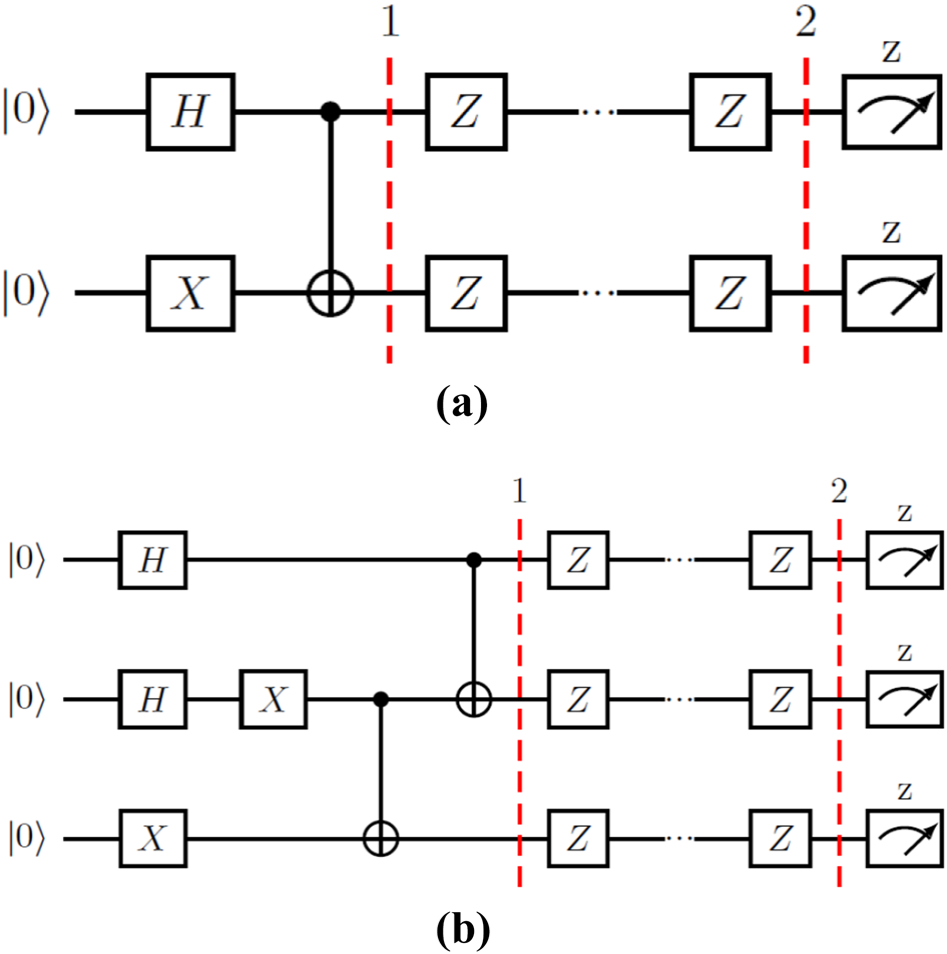
Circuits for the initialization and application of a two-qubit (**a**) and three-quibit (**b**) LEO, through *Z* gates. At step 1, we have successfully initialized the system in the desired state, and step 2 means the application of the LEO has finished.

The first two LEOs we tested consist of a sequence of *Z* gates applied to all qubits in the system, which is the first example presented in^2^. The protected subspace for the two-qubit $(d=2)$ case is $\{|{01}\rangle ,|{10}\rangle \}$ with $R_{L}^{d}=Z_1Z_2$; in the three-qubit $(d=3)$ case, the protected subspace is $\{|{001}\rangle , |{010}\rangle , |{100}\rangle , |{111}\rangle \}$ and $R_{L}^{d}=Z_1Z_2Z_3$. In these subspaces, the application of a *Z* operator for each qubit follows the form given in (8) with $\hat{n}=\hat{u}_{z}$; acting as *I* in the protected subspace $\mathcal {S}_{1}$ or $\mathcal {S}_{2}$ and as $-I$ in its orthogonal subspace. For our tests, we have chosen initial states with equal populations in every state of the protected subspace: $|{\Psi _{2}}\rangle =(|{01}\rangle +|{10}\rangle )/\sqrt{2}$ and $|{\Psi _{3}}\rangle =(|{001}\rangle +|{010}\rangle +|{100}\rangle +|{111}\rangle )/2$, respectively. The gates applied for initialization and LEO application can be seen in the circuits in Fig. 1a,b.

The third example considers how a CNOT gate behaves as an LEO. In this case, the subspace spanned by $|{10}\rangle -|{11}\rangle$, $\mathcal {S}_{3}$, is protected from leaking into $\{|{00}\rangle , |{01}\rangle , |{10}\rangle +|{11}\rangle \}$. The effectiveness of CNOT as an LEO in this system is easy to see if we write its explicit matrix form in the basis of $\{|{00}\rangle , |{01}\rangle , |{10}\rangle +|{11}\rangle ,|{10}\rangle -|{11}\rangle \}$:

10 $$\begin{aligned} \text {CNOT}=\begin{pmatrix} 1 &{} 0 &{} 0 &{} 0 \\ 0 &{} 1 &{} 0 &{} 0 \\ 0 &{} 0 &{} 1 &{} 0 \\ 0 &{} 0 &{} 0 &{} -1 \end{pmatrix} \end{aligned}$$

**Figure 2:**
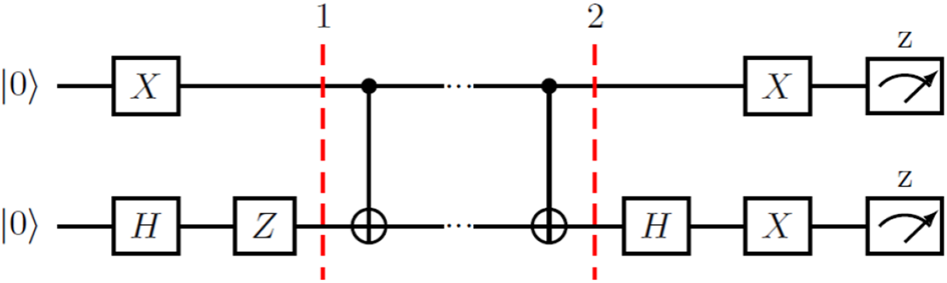
Circuit for the initialization and application of a two-quibit LEO, through CNOT gates. At step 1, we have successfully initialized the system in the desired state, and step 2 means the application of the LEO has finished. Note that after the application of the LEO, we must further manipulate our system for measurement purposes.

This is in accordance with the form of a universal LEO given in (5), meaning that CNOT is an LEO in the chosen basis. We prepare the initial state $|{\Phi }\rangle =(|{10}\rangle -|{11}\rangle )/\sqrt{2}$ when studying this LEO. Since the measurement options in IBM’s cloud computer are limited to measuring the *z* component of the spin of the qubits, further manipulation of the system has been needed for measurement purposes − that is, to be able to discern what part of the populations of $|{10}\rangle$ and $|{11}\rangle$ correspond to the protected subspace $\{|{10}\rangle -|{11}\rangle \}$. After the LEO has been applied *N* times, we have applied a Hadamard gate to the second qubit, and then an *X* gate to both qubits, so that $|{\Phi }\rangle$ turns into $|{00}\rangle$ before the measurement. The circuit describing this can be seen in Fig. 2.

Once we have completed all runs, we compare the fidelity of the run where an LEO was applied with the one achieved through free evolution. This quantity is obtained for each *N*, by computing the ratio of the populations of the desired states by the number of total pulses in each run. For example, in the case of the two-qubit system where we used *Z* as an LEO, this fidelity would be $f_{1}={(\text {number of } |{01}\rangle \text { states} + \text {number of } |{10}\rangle \text { states})}/{1024}$.

## Results and discussion

**Figure 3:**
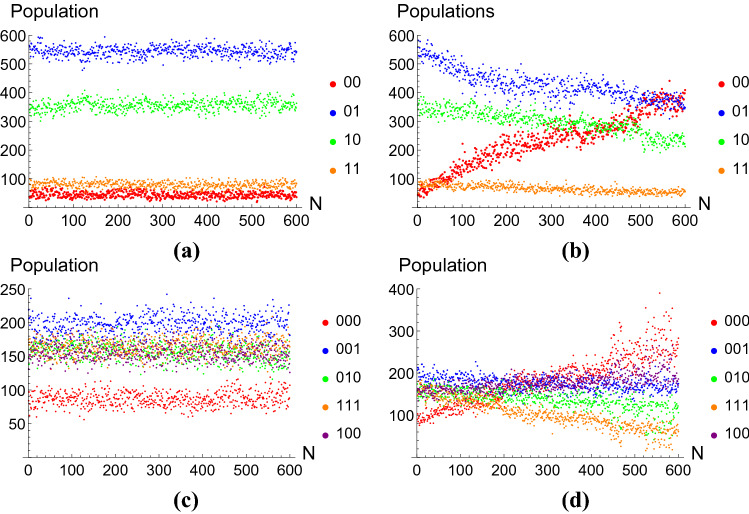
Populations of individual states measured at different times, corresponding to up to 600 pulses, with he application of Z as an LEO in (**a**,**c**), and with free evolution in (**b**,**d**). (**a**,**b**) Show the populations of $|{00}\rangle ,|{01}\rangle ,|{10}\rangle \ \text {and} \ |{11}\rangle$, while (**c**,**d**) show the populations of $|{000}\rangle$, $|{001}\rangle$, $|{010}\rangle$, $|{111}\rangle$ and $|{100}\rangle$ (that is, the populations of the protected subspace states). At each time, 1024 shots where fired.

Figure 3 shows the population of each state in the cases where *Z* was used as an LEO. We can clearly see that, when an LEO is used (see Fig. 3a,c), initial populations remain roughly constant over time, with some expected fluctuations due to the probabilistic nature of quantum mechanics. While one would expect populations of the components of the initial state to be evenly distributed [for example, having equal populations of $|{01}\rangle$ and $|{10}\rangle$ in Fig. 3a, differences in the physical qubits prevent this from happening^25^. When the LEO is not applied (Fig. 3b,d), the population of the ground state increases, as the population of the states of interest decreases. This is due to the decay of qubits in state $|{1}\rangle$ into the lower energy state $|{0}\rangle$. This is precisely the leakage we seek to prevent through the use of LEOs.

**Figure 4:**
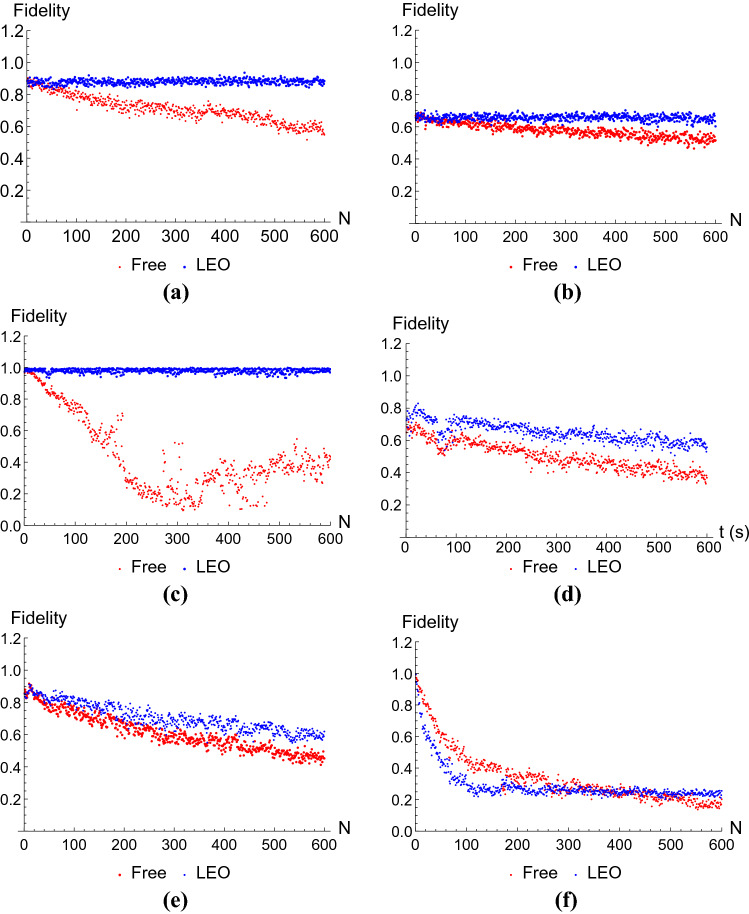
Fidelity of quantum states measured at different times, corresponding to up to 600 pulses, with 1024 shots at each time. The blue dots correspond to the run where LEO pulses where applied; the red dots correspond to the freely evolving system. (**a**,**b**) Show the evolution of a two-qubit system, with inital state $|{\Psi _{2}}\rangle =(|{01}\rangle +|{10}\rangle )/\sqrt{2}$ and Z pulses used as an LEO, with (**b**) having identity pulses applied between LEO applications; (**c**,**d**) depict the three-qubit case that also uses Z as an LEO, with initial state $|{\Psi _{3}}\rangle =(|{001}\rangle +|{010}\rangle +|{100}\rangle +|{111}\rangle )/2$, again, (**d**) has had identity pulses applied between LEO applications. In (**e**,**f**) we used $|{\Phi }\rangle =(|{10}\rangle -|{11}\rangle )/\sqrt{2}$ as our initial state and the role of LEO was carried out by CNOT gates, having identity pulses applied between LEO applications for (**f**).

**Figure 5:**
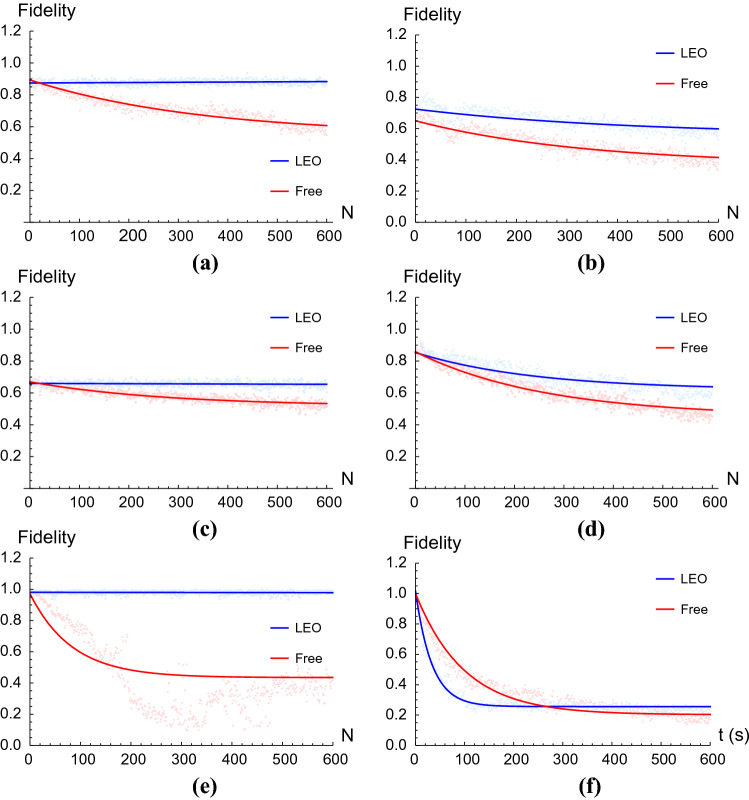
Curves fitted to the fidelity of quantum states measured at different times, corresponding to up to 600 pulses, with 1024 shots at each time. The blue curves correspond to the run where LEO pulses where applied, and have been fitted with Eq. (11) for (**a**,**c**,**e**) and Eq. (12) for (**b**,**d**,**f**). The red curves correspond to the freely evolving systems and have all been fitted with the exponential function in (12). (**a**,**b**) Show the evolution of a two-qubit system, with inital state $|{\Psi _{2}}\rangle =(|{01}\rangle +|{10}\rangle )/\sqrt{2}$ and Z pulses used as an LEO, with (**b**) having identity pulses applied between LEO applications; (**c**,**d**) depict the three-qubit case that also uses Z as an LEO, with initial state $|{\Psi _{3}}\rangle =(|{001}\rangle +|{010}\rangle +|{100}\rangle +|{111}\rangle )/2$, again, (**d**) has had identity pulses applied between LEO applications. In (**e**,**f**) we used $|{\Phi }\rangle =(|{10}\rangle -|{11}\rangle )/\sqrt{2}$ as our initial state and the role of LEO was carried out by CNOT gates, having identity pulses applied between LEO applications for (**f**).

We have found that the performance of the LEOs varies between operators. It also depends on the dimension of the studied system. All experiments, however, share one trait: application of an LEO maintains the fidelity of the final state constant in time, as we can see in Fig. 4. This, in accordance to Fig. 3a,c, means the population of states in the protected subspace remains constant—that is, *protected*. To quantify this, we have fitted several curves to the gathered data through Mathematica, obtaining what can be seen in Fig. 5. Since we expect LEOs to fully protect the system from leakage and we thus anticipate that the initial fidelity will remain constant in time, we have chosen a linear fit for the cases in which the LEO was applied optimally, that is, Fig. 5a,c,e:

11 $$\begin{aligned} f_{i}^{\text {lin}}(a_{i},b_{i};N)=a_{i}+b_{i}(N-1), \quad i=1,2,3 \end{aligned}$$

The obtained parameters and their standard deviations can be seen in Table 1. The rest of the curves have been fitted to an exponential function of the form

12 $$\begin{aligned} f_{i}^{\text {exp}}(\alpha _{i},\beta _{i},\gamma _{i};N)=\alpha _{i}+\beta _{i}e^{-\gamma _{i}(N-1)}, \quad i=1,2,3\ , \end{aligned}$$

where we choose $\beta _{i}$ the fidelity at $N=1$ and $\alpha _{i}$ to be the fidelity at $N\rightarrow \infty$. In practice, we have imposed $\alpha _{i}=f_{i}^{\text {exp}}(N=600)$, since it is the last piece of information we have. In any case, this is an optimistic choice, since we expect exponential decay to continue after our last measurement. The values we obtained for these parameters are collected in Tables 2, 3 and 4.

**Table 1:** Parameters obtained after fitting the measured fidelities of systems prepared in states $|{\Psi _{1}}\rangle =(|{01}\rangle +|{10}\rangle )/\sqrt{2}$, $|{\Psi _{2}}\rangle =(|{001}\rangle +|{010}\rangle +|{100}\rangle +|{111}\rangle )/2$ and $|{\Psi _{3}}\rangle =(|{001}\rangle +|{010}\rangle +|{100}\rangle +|{111}\rangle )/2$ after application of $N\in [1,600]$ LEOs to the linear function in Eq. (11).

	$a_{i}$	$b_{i}$
$f_{1}^{\text {lin}}$	$(8.75\pm 0.01) \times 10^{-1}$	$(1.4\pm 0.4) \times 10^{-5}$
$f_{2}^{\text {lin}}$	$(6.60\pm 0.01)\times 10^{-1}$	$(1.0\pm 0.4)\times 10^{-5}$
$f_{3}^{\text {lin}}$	$(9.81\pm 0.01)\times 10^{-1}$	$(-3\pm 3)\times 10^{-6}$

The specific LEOs we used are *Z* for $|{\Psi _{1}}\rangle$ and $|{\Psi }\rangle _{2}$ and CNOT for $|{\Psi _{3}}\rangle$.

**Table 2:** Parameters obtained after fitting the measured fidelities of systems prepared in states $|{\Psi _{1}}\rangle =(|{01}\rangle +|{10}\rangle )/\sqrt{2}$, $|{\Psi _{2}}\rangle =(|{001}\rangle +|{010}\rangle +|{100}\rangle +|{111}\rangle )/2$ and $|{\Psi _{3}}\rangle =(|{001}\rangle +|{010}\rangle +|{100}\rangle +|{111}\rangle )/2$ after application of $N\in [1,600]$ identity gates (free evolution) to the exponential function in Eq. (12).

	$\alpha _{i}$	$\beta _{i}$	$\gamma _{i}$
$f_{1}^{\text {exp}}$	0.546875	0.345703	$(2.91\pm 0.03) \times 10^{-3}$
$f_{2}^{\text {exp}}$	0.515625	0.15332	$(3.61\pm 0.06)\times 10^{-3}$
$f_{3}^{\text {exp}}$	0.43457	0.53125	$(1.20\pm 0.08) \times 10^{-2}$

**Table 3:** Parameters obtained after fitting the measured fidelities of systems prepared in states $|{\Psi _{1}}\rangle =(|{01}\rangle +|{10}\rangle )/\sqrt{2}$, $|{\Psi _{2}}\rangle =(|{001}\rangle +|{010}\rangle +|{100}\rangle +|{111}\rangle )/2$ and $|{\Psi _{3}}\rangle =(|{001}\rangle +|{010}\rangle +|{100}\rangle +|{111}\rangle )/2$ after application of $N\in [1,600]$ LEOs to the exponential function in Eq. (12), with extra identity gates between LEO applications.

	$\alpha _{i}$	$\beta _{i}$	$\gamma _{i}$
$f_{1}^{\text {exp}}$	0.557617	0.166992	$(2.35\pm 0.07)\times 10^{-3}$
$f_{2}^{\text {exp}}$	0.619141	0.233398	$(4.16\pm 0.09)\times 10^{-3}$
$f_{3}^{\text {exp}}$	0.255859	0.738281	$(3.00\pm 0.04)\times 10^{-2}$

The specific LEOs we used are *Z* for $|{\Psi _{1}}\rangle$ and $|{\Psi }\rangle _{2}$ and CNOT for $|{\Psi _{3}}\rangle$.

**Table 4:** Parameters obtained after fitting the measured fidelities of systems prepared in states $|{\Psi _{1}}\rangle =(|{01}\rangle +|{10}\rangle )/\sqrt{2}$, $|{\Psi _{2}}\rangle =(|{001}\rangle +|{010}\rangle +|{100}\rangle +|{111}\rangle )/2$ and $|{\Psi _{3}}\rangle =(|{001}\rangle +|{010}\rangle +|{100}\rangle +|{111}\rangle )/2$ after application of $N\in [1,600]$ pairs of identity gates (extended free evolution) to the exponential function in Eq. (12).

	$\alpha _{i}$	$\beta _{i}$	$\gamma _{i}$
$f_{1}^{\text {exp}}$	0.369141	0.280273	$(3.01\pm 0.05)\times 10^{-3}$
$f_{2}^{\text {exp}}$	0.452148	0.404297	$(3.82\pm 0.04) \times 10^{-3}$
$f_{3}^{\text {exp}}$	0.202148	0.790039	$(1.01 \pm 0.01)\times 10^{-2}$

Taking a look at Table 1, we are able to quantify the efficiency of LEOs. The linear fitting of the data confirms what we already suspected from Fig. 4a–c: the fidelity of the protected states remains nearly constant, the $b_{i}$ being at least four orders of magnitude smaller than all $a_{i}$. In fact, we would expect $b_{i}<0$ for all cases, however, we only get this for $b_{3}$, which has an standard error of the same magnitude and even value as the parameter itself. The standard errors of the $a_{i}$, however, allow for three significant figures in the parameters. This difference in precision, together with our knowledge that the fidelity of this states should decrease in time, makes the accuracy of the $b_{i}$ dubious, and we will thus consider the fidelity constant from now on, taking the values given by $a_{i}$.

To compare these results to the freely evolving case, we will compare the expected fidelities at $N\rightarrow \infty$. For the system protected by a LEO, this will be $a_{i}$, as we have previously justified. For the freely evolving systems, we have chosen $\alpha _{i}$ to be the fidelity at $N\rightarrow \infty$, even if, as has already been stated, this actually corresponds to the fidelity at $N=600$. We must thus keep in mind that we weill be dealing with an optimistic prediction for the expected fidelity at $N\rightarrow \infty$ for the freely evolving case.

When *Z* gates were used as an LEO, we have achieved $f_{1}^{\text {LEO}}(N\rightarrow \infty )\approx a_{1}=(8.75\pm 0.01)\times 10^{-1}$ for the two-qubit case, and $f_{2}^{\text {LEO}}(N\rightarrow \infty )\approx a_{2}=(6.60\pm 0.01)\times 10^{-1}$ for the three qubit case. Compared to their freely evolving counterparts of $f_{1}^{\text {Free}}(N\rightarrow \infty )\approx \alpha _{1}=5.47\times 10^{-1}$ and $f_{2}^{\text {Free}}(N\rightarrow \infty )\approx \alpha _{2}=5.16\times 10^{-1}$, this poses a clear improvement, specially if we remember that we should expect an even lower fidelity at $N\rightarrow \infty$ for the freely evolving case. The improvement is even more so notorious for the system where we applied CNOT gates as an LEO, with $f_{3}^{\text {LEO}}(N\rightarrow \infty )\approx a_{3}=(9.81\pm 0.01)\times 10^{-1}$ and $f_{3}^{\text {Free}}(N\rightarrow \infty )\approx \alpha _{1}=4.35\times 10^{-1}$. This greater efficiency may be a consequence of the presence of $|{00}\rangle$ states prior to the manipulation of the final state for measurement purposes. The improvement in fidelity is, nonetheless, the most significant among our three cases of study.

On another note, looking at Fig. 4d–f, we can see that an increase in free evolution time between LEO pulses decreases the effectiveness of leakage elimination. This is to be expected, since, as explained in “Universal leakage elimination”, the ideal LEO relies on BB pulses for effectiveness. That is, elongating the time between pulses strays us further away from the ideal LEO. This effect is the most prominent for the case of CNOT gates (see Fig. 4f), which makes the application of an LEO counterproductive. These data have been fitted to exponential curves of the form (12), and the obtained parameters can be seen in Table 3, for the case where LEOs were applied, and (4), for the freely evolving cases. We see that the fidelity achieved at $N\rightarrow \infty$ is lower than when LEOs were applied with no identity gates between pulses, with $f_{1}^{\text {LEO+ID}}(N\rightarrow \infty )\approx \alpha _{1}=5.58\times 10^{-1}$, $f_{2}^{\text {LEO+ID}}(N\rightarrow \infty )\approx \alpha _{2}=6.19\times 10^{-1}$ and $f_{3}^{\text {LEO+ID}}(N\rightarrow \infty )\approx \alpha _{3}=2.56\times 10^{-1}$.

## Conclusion

The present experiments confirm that LEOs are able to help stabilize fidelity for two- and three-qubit leakage-free subspaces on IBMQ’s 5-qubit QC, as predicted in our theories. The three subspaces are decoherence-free against collective dephasing, for three-site quantum state transfer and eigenvectors of the CNOT gate. Although IBMQ’s 5-qubit QC limits the application of ideal dynamic decoupling control sequences, further decay in fidelity has been successfully suppressed, confirming that the LEO time-scale condition can be satisfied with noise in the device. Figure 4f shows a counterexample, where the LEO time-scale condition is *not* satisfied with noise in the device. Therefore, as long as the time interval between pulse applications is short enough (within the capability of IBMQ’s 5-qubit QC), the use of an LEO is surely advantageous with respect to a free evolution of the system. Keeping in mind that LEOs are universal and can be applied alongside other qubit operations in the corresponding code space, we have experimentally provided a powerful tool for higher accuracy of quantum algorithms.
